# A New Method for Albuminuria Measurement Using a Specific Reaction between Albumin and the Luciferin of the Firefly Squid *Watasenia scintillans*

**DOI:** 10.3390/ijms23158342

**Published:** 2022-07-28

**Authors:** Tetsuya Ishimoto, Takuya Okada, Shiho Fujisaka, Kunimasa Yagi, Kazuyuki Tobe, Naoki Toyooka, Hisashi Mori

**Affiliations:** 1 Department of Molecular Neuroscience, Faculty of Medicine, University of Toyama, 2630 Sugitani, Toyama City, Toyama 930-0194, Japan; hmori@med.u-toyama.ac.jp; 2 Research Center for Idling Brain Science, University of Toyama, 2630 Sugitani, Toyama City, Toyama 930-0194, Japan; 3 Faculty of Engineering, University of Toyama, 3190 Gofuku, Toyama City, Toyama 930-8555, Japan; tokada@eng.u-toyama.ac.jp (T.O.); toyooka@eng.u-toyama.ac.jp (N.T.); 4 First Department of Internal Medicine, Faculty of Medicine, University of Toyama, 2630 Sugitani, Toyama City, Toyama 930-0194, Japan; shihof@med.u-toyama.ac.jp (S.F.); yagikuni@icloud.com (K.Y.); tobe@med.u-toyama.ac.jp (K.T.); 5 Department of Internal Medicine, Kanazawa Medical University, 1-1 Daigaku, Uchinada-machi, Kahoku-gun, Ishikawa 920-0293, Japan; 6 Research Center for Pre-Disease Science, University of Toyama, 2630 Sugitani, Toyama City, Toyama 930-0194, Japan

**Keywords:** *Watasenia scintillans*, luciferin, albumin, bioluminescence, urine, diabetic nephropathy

## Abstract

This study demonstrates that the luciferin of the firefly squid *Watasenia scintillans*, which generally reacts with *Watasenia* luciferase, reacted with human albumin to emit light in proportion to the albumin concentration. The luminescence showed a peak wavelength at 540 nm and was eliminated by heat or protease treatment. We used urine samples collected from patients with diabetes to quantify urinary albumin concentration, which is essential for the early diagnosis of diabetic nephropathy. Consequently, we were able to measure urinary albumin concentrations by precipitating urinary proteins with acetone before the reaction with luciferin. A correlation was found with the result of the immunoturbidimetric method; however, the *Watasenia* luciferin method tended to produce lower albumin concentrations. This may be because the *Watasenia* luciferin reacts with only intact albumin. Therefore, the quantification method using *Watasenia* luciferin is a new principle of urinary albumin measurement that differs from already established methods such as immunoturbidimetry and high-performance liquid chromatography.

## 1. Introduction

The firefly squid *Watasenia scintillans* is a luminescent deep-sea squid that inhabits the Japan Sea. As it migrates from the deep sea to shallow waters to spawn every spring, the blue luminescence can be observed by the naked eye. The brightest light organ of *Watasenia* is located at the tip of the fourth arm that emits light. Other small organs scattered on the mantle emit weaker light. The luminescence is considered a counter-illumination system to escape from predators [1]. Like other luminescent organisms, *W. scintillans* has luciferase and luciferin in its body [2,3]. The luciferase of *Watasenia* has been found to be ATP-dependent and to exist in a crystalline structure [4,5]. In recent years, there have been attempts to identify *Watasenia* luciferase through mass spectrometry and transcriptome analysis; however, active luciferase has not yet been purified [6,7]. *Watasenia* luciferin has been identified and isolated from the light organ in arm photophores [8,9,10]. The structure of *Watasenia* luciferin is similar to that of coelenterazine, which is a luciferin present in a wide variety of marine luminescent organisms [11,12]. Although there are no reports of *Watasenia* luciferin being used for testing in medicine, this paper explores the potential use of the luciferin in medical testing.

The kidney has a functional component unit involved in urine production called the nephron, which is composed of the glomerulus and tubules, and it is known that albumin leaks into the urine when the glomerulus is damaged. Albuminuria is considered a risk factor for various diseases, among which diabetic nephropathy is known to be closely related to urinary albumin concentration. Diabetic nephropathy is a complication of diabetes and progresses through several stages. In its early stage, trace amounts of albumin are leaked into the patient’s urine. Early diagnosis and appropriate glycemic and blood pressure control improve the prognosis of diabetic nephropathy [13,14,15,16,17]. Therefore, measuring urinary albumin levels is critical for suppressing diabetic nephropathy progression. Urinary albumin concentrations are very low compared with serum albumin concentrations, and they coexist with several molecules. Moreover, urine samples contain intact as well as half-degraded albumin, which is not observed in blood samples. These factors complicate the measurement of urinary albumin and the interpretation of its results.

The measurement of urinary albumin by dipstick is simple and rapid, but it is not highly quantitative. Immunoturbidimetry is a method used to quantify antigen–antibody complex precipitates in a liquid by measuring the optical density, and it is widely used to measure albumin concentrations in the urine of patients [18]. Although this method is simple and sensitive, methods based on antigen–antibody reactions exhibit a prozone (hook) effect, in which the signal is reversed at very high albumin concentrations [19]. Furthermore, several researchers have reported the presence of immuno–unreactive albumin in urine [20]. In methods based on high-performance liquid chromatography (HPLC), albumin is purified according to its molecular weight, and its concentration is measured; these methods are considered to be capable of measuring immuno–unreactive albumin [21]. Nevertheless, HPLC-based methods can also measure proteins of the same molecular weight as that of albumin. Therefore, HPLC-based methods tend to produce larger albumin concentrations than antibody-based methods [22]. In this study, we report that *Watasenia* luciferin reacts with human albumin and emits luminescence; we also attempt to establish the *Watasenia* luciferin method as a new quantitative technique for measuring urinary albumin levels in addition to the two major existing methods.

## 2. Results

### 2.1. Specific Reaction and Luminescence of Watasenia Luciferin and Human Albumin

**Scheme 1:**
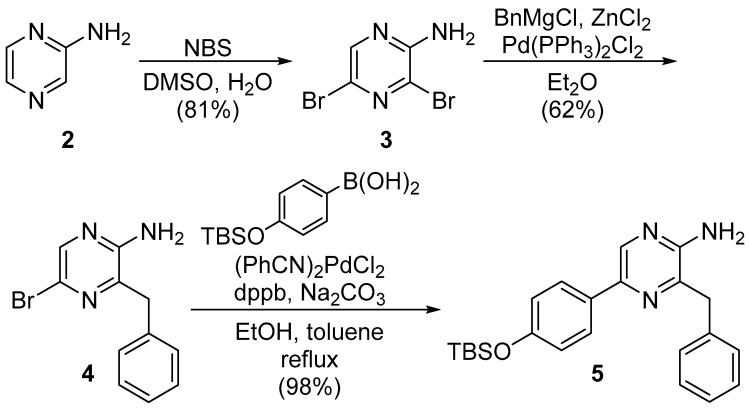
Synthesis of the 3,5-disubstituted pyrazin-2-amine 5.

**Scheme 2:**
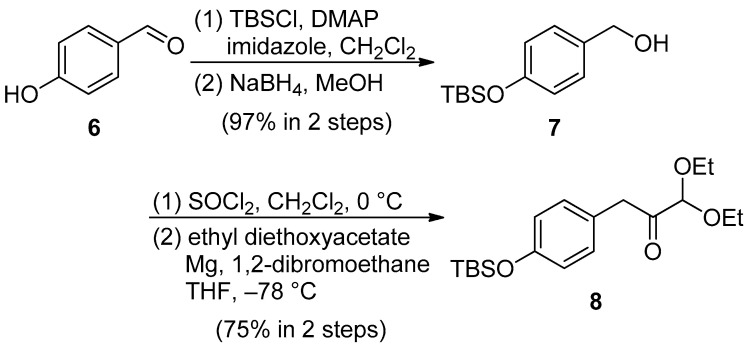
Synthesis of the acetal derivative **8**.

**Scheme 3:**
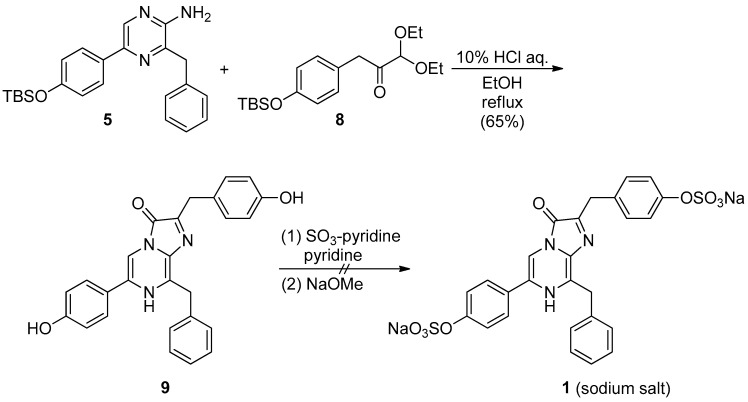
Synthesis of the sodium salt of *Watasenia* luciferin **1**.

**Scheme 4:**
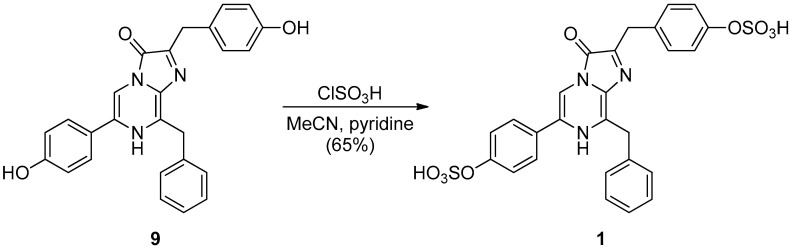
Synthesis of *Watasenia* luciferin **1**.

We synthesized *Watasenia* luciferin according to a previously reported method [23], with a slight modification (Scheme 1, Scheme 2, Scheme 3 and Scheme 4 and Supplementary Figures S1 and S2). We first planned to isolate *Watasenia* luciferase using the synthesized luciferin, but this was not successful. However, in this process, we discovered by chance that *Watasenia* luciferin reacted with albumin and emitted light, and then we began characterizing the albumin–luciferin reaction.

**Figure 1:**
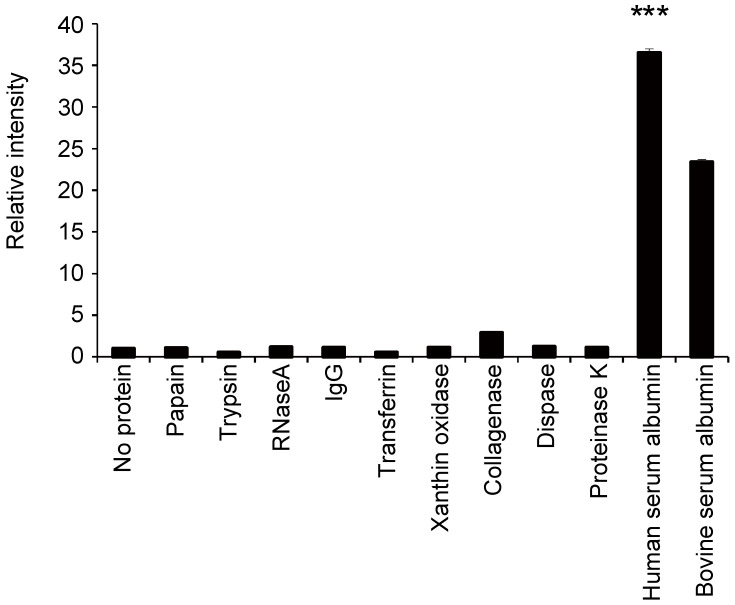
Relative luminescence intensity emitted by the reaction of each protein (1 mg/mL) (horizontal axis) with *Watasenia* luciferin. The values for each group were compared with human albumin values (mean ± SEM, *n* = 4; one-way ANOVA with Dunnett’s test, *** *p* < 0.001).

Human albumin, bovine albumin, and several other proteins were mixed with *Watasenia* luciferin, and their luminescence was measured (Figure 1). The strongest luminescence was observed in the reaction with human albumin, and the intensities of all other groups were compared against human albumin to determine whether the difference was significant. The luminescence levels of other proteins were similar to those observed without the protein. This indicates that luciferin reacts specifically with albumin and emits luminescence.

### 2.2. Optimization of the Albumin–Luciferin Reaction

**Figure 2:**
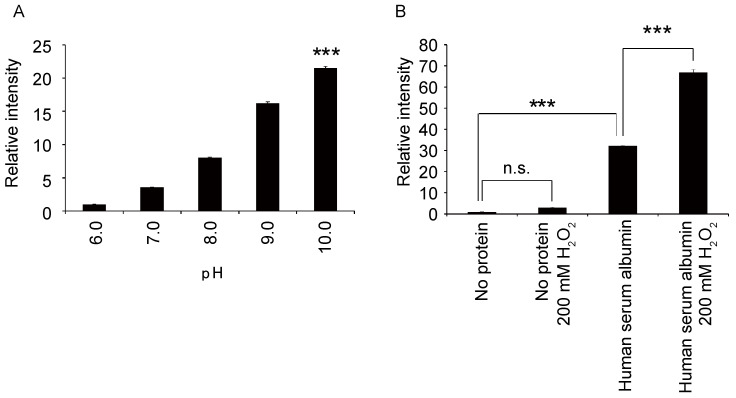
(**A**) Relative changes in the luminescence intensity of the albumin–luciferin reaction at each pH value (80 mM, Tris) (mean ± SEM, *n* = 4; one-way ANOVA with Dunnett’s test, *** *p* < 0.001). (**B**) Relative changes in the luminescence intensity of the albumin–luciferin reaction with or without 200 mM hydrogen peroxide (mean ± SEM, *n* = 4; one-way ANOVA with Tukey’s test, *** *p* < 0.001, n.s. = not significant).

To optimize the albumin–luciferin reaction, Tris buffer solutions with several pH values in the reaction solution were evaluated. The strongest luminescence was observed when the reaction was performed using a buffer with pH 10 (Figure 2A). As the luminescence of luciferin is considered to be due to oxidation [24], we evaluated whether the addition of H_2_O_2_ to the solution would change the luminescence level. The results showed that the luminescence increased with the addition of albumin, even without H_2_O_2_; however, in the presence of H_2_O_2_, the luminescence increased even further (Figure 2B). Based on these findings, we measured the luminescence with H_2_O_2_ in a buffer solution with pH 10.

### 2.3. Characterization of Albumin–Luciferin Bioluminescence

**Figure 3:**
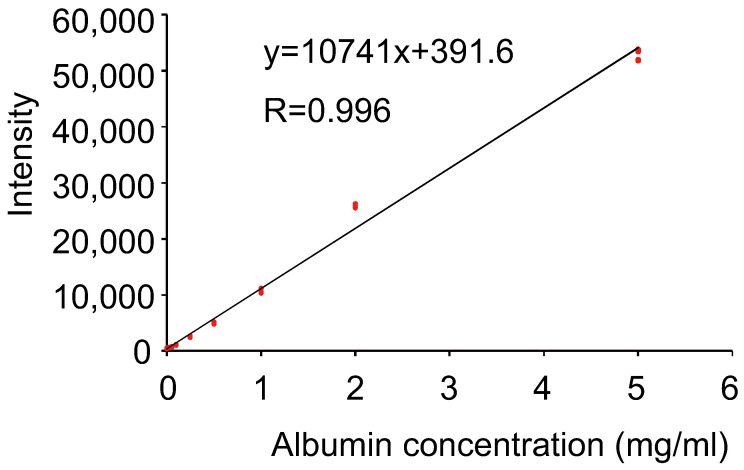
Scatter plots (red mark) and the approximate straight line of albumin concentration versus luminescence intensity (*n* = 4 in each dose).

**Figure 4:**
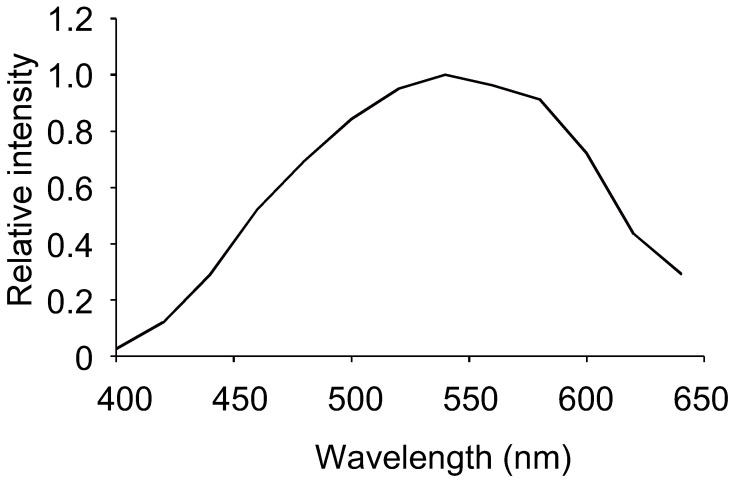
Luminescence spectrum of the albumin–luciferin reaction.

We investigated the relationship between the concentration of human albumin and the amount of luminescence. Luminescence exhibited a proportional relationship with the concentration of human albumin within the concentration range of 0−5 mg/mL (Figure 3). The correlation coefficient was 0.996, and the calculated detection limit was 20 μg/mL. The spectrum of the reaction between human albumin and *Watasenia* luciferin was measured and was found to peak at 540 nm, which differed from the blue luminescence of living *W. scintillans* (Figure 4).

**Figure 5:**
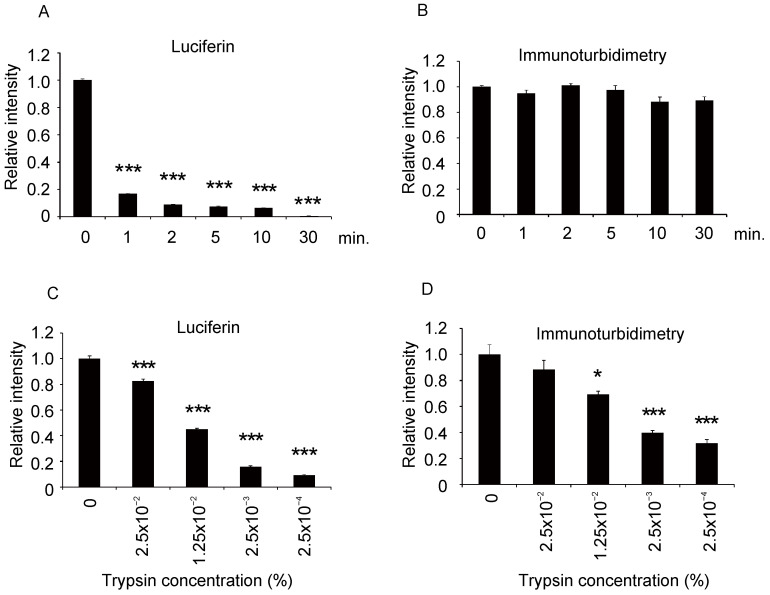
Effect of heat denaturation of albumin and trypsin treatment on albumin–luciferin luminescence and immunoturbidimetric signals. Purified human albumin was heat-denatured at 95 °C for each time period, and then the relative reduction in signals was measured by (**A**) albumin–luciferin reaction and (**B**) immunoturbidimetric assays. Similarly, the albumin level was measured after trypsin treatment (**C**,**D**). The values for each group were compared with the values of 0 min or 0% (mean ± SEM, *n* = 4; one-way ANOVA with Dunnett’s test, * *p* < 0.05, *** *p* < 0.001).

We then determined the manipulations that would inhibit the luminescence to characterize the albumin–luciferin reaction. When the human albumin was heat-denatured, the amount of luminescence decreased depending on the duration of heat treatment (Figure 5A). This indicates that albumin loses its luminescence ability when its higher-order structure is destroyed by thermal denaturation. However, the signal in the immunoturbidimetric assay showed no significant decrease, even with the albumin that was heat-denatured for 30 min (Figure 5B).

When the albumin was treated with different concentrations of trypsin and a serine protease and reacted with luciferin, the amount of luminescence decreased according to the trypsin concentration (Figure 5C). This indicates that the partial cleavage of the peptide by trypsin leads to the loss of luminescence ability. When the same trypsinized samples were measured through the immunoturbidimetric assay, the signal decreased depending on the trypsin concentration, but the decrease was milder than that in the reaction with luciferin (Figure 5D). These results indicate that the immunoturbidimetric method is more resistant to the partial cleavage of albumin than the albumin–luciferin reaction.

### 2.4. Detection of Albumin in the Urine of Diabetic Patients Using the Albumin–Luciferin Reaction

**Figure 6:**
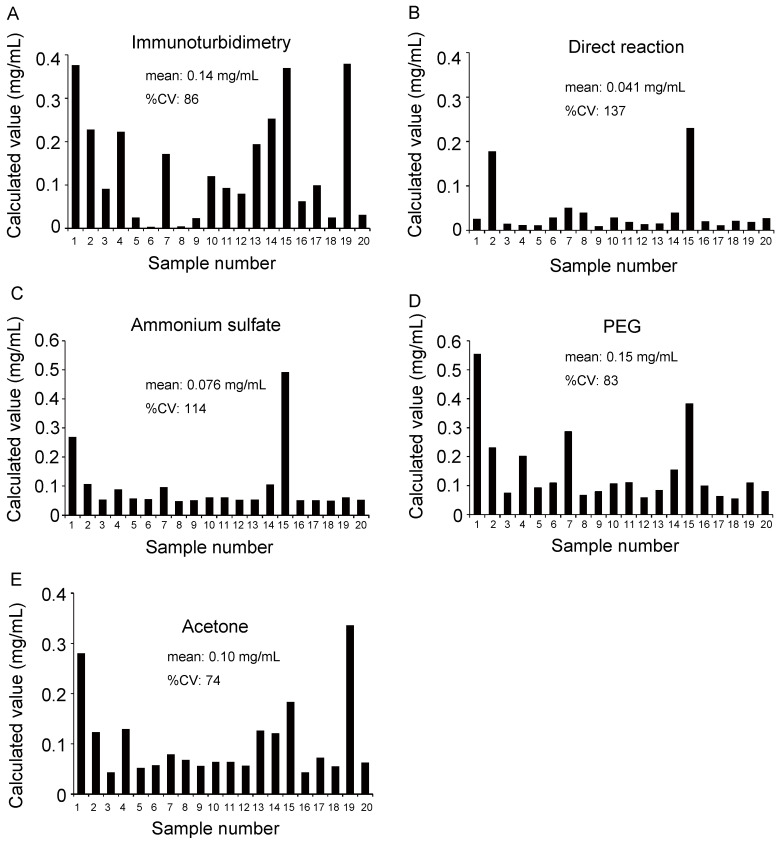
Comparison of albumin concentrations quantified by various methods. (**A**) Urine sample was quantified by an immunoturbidimetric assay. (**B**) Urine samples and albumin purified by (**C**) ammonium sulfate precipitation, (**D**) polyethylene glycol (PEG) precipitation, and (**E**) acetone precipitation were reacted with luciferin, and albumin concentration was calculated based on luminescence. The horizontal axis represents the sample number, and the vertical axis represents the calculated albumin concentration.

**Figure 7:**
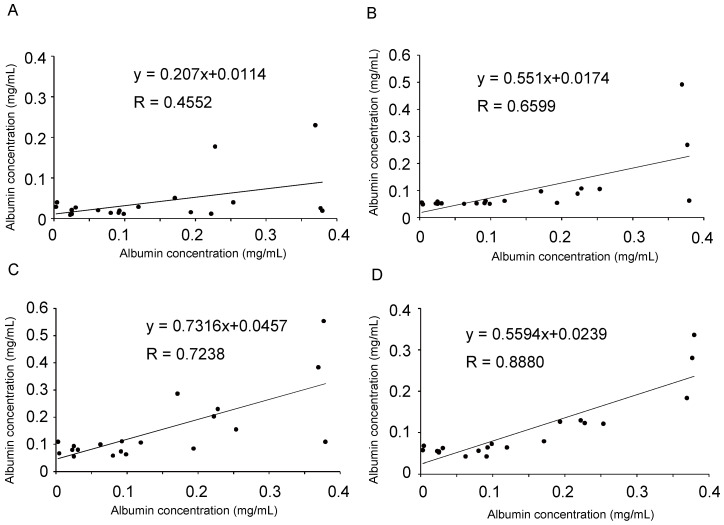
Comparison of albumin quantification results obtained from the immunoturbidimetric assay with those obtained using the albumin–luciferin reaction. Based on the values calculated in Figure 6, correlations were determined between (**A**) immunoturbidimetry versus the direct reaction of urine with luciferin, (**B**) immunoturbidimetry versus ammonium sulfate precipitation, (**C**) immunoturbidimetry versus PEG precipitation, and (**D**) immunoturbidimetry versus acetone precipitation. In each panel, the horizontal axis indicates the value of the immunoturbidimetric method. The approximate straight line and correlation coefficient were calculated.

Next, because measuring albumin levels in urine is an essential method for the diagnosis of diabetic nephropathy, we evaluated the feasibility of using luciferin to measure trace albumin levels in the urine of 20 patients with diabetes. First, we reacted the urine sample directly with luciferin and measured the luminescence. However, the quantitative results from the direct reaction between urine and luciferin revealed a low correlation with the immunoturbidimetric results (Figure 6A,B and Figure 7A). This may be because coexisting molecules in urine inhibit the albumin–luciferin reaction. Next, we attempted to purify the protein in urine and react it with albumin to remove foreign substances in the urine. For this purpose, we used ammonium sulfate precipitation, polyethylene glycol (PEG) precipitation, and acetone precipitation.

**Figure 8:**
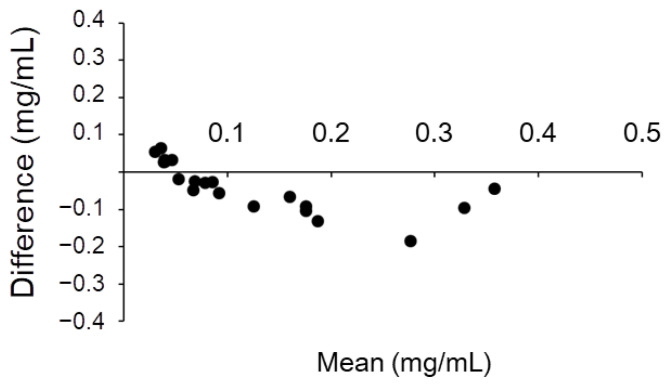
Bland–Altman plot of the values of immunoturbidimetry and the luciferin method after acetone precipitation. The horizontal axis represents the mean of the two methods, and the vertical axis represents the value of the luciferin method minus that of the immunoturbidimetric method.

In each method, the urine sample and standard albumin were precipitated, dissolved again in phosphate-buffered saline (PBS), and reacted with luciferin. The concentration of albumin was calculated and graphed based on the intensity of luminescence (Figure 6C–E). The results were compared with those of the immunoturbidimetric assay (Figure 6A) using urine samples; scatter plots were prepared, and correlation coefficients were calculated (Figure 7B–D). Particularly, we observed a high correlation when acetone was used to precipitate the protein in the urine (Figure 7D). Moreover, each purification method showed a lower value than the albumin quantitative value obtained using the immunoturbidimetric method (Figure 7A–D). We analyzed the data used in Figure 7D again using the Bland–Altman plot. In urine containing high concentrations of albumin, the luciferin method tended to yield lower readings than immunoturbidimetry (Figure 8). The coefficient of variation (CV) value from the repeated measurements of luminescence from acetone-precipitated samples of the same concentration was 5.2% (*n* = 8). In addition, a spike and recovery test was performed, and it was found that the recovery rate was 105%. This result means that the component in urine did not inhibit the reaction.

## 3. Discussion

This study demonstrated that *Watasenia* luciferin specifically reacted with albumin and emitted light (Figure 1). When reacted with purified albumin, it exhibited a very obvious proportional relationship (Figure 3). Since the calculated detection limit (20 μg/mL) was lower than the range of microalbuminuria (30–300 μg/mL), *Watasenia* luciferin has a sufficient measurement range and sensitivity to measure trace albuminuria. This is a new discovery and indicates that *Watasenia* luciferin can be used to measure albumin concentrations in liquids. Moreover, after exploring the optimal conditions for the reaction, 200 mM H_2_O_2_ at pH 10 was found to be the best (Figure 2). This result is reasonable because it has been previously shown that oxygen is required for the luminescence of *W. scintillans* [24].

An earlier report described that coelenterazine, a molecule similar to *Watasenia* luciferin, reacts with albumin to emit light [25]. Although the underlying mechanism was not confirmed, it was predicted that Sudlow site I, to which molecules such as warfarin bind [26], would be the binding site for coelenterazine based on the three-dimensional structure of albumin. *Watasenia* luciferin may have a similar mechanism of action. *Watasenia* luciferin reacted more strongly with human albumin than with bovine albumin, which is in contrast to the action of coelenterazine [25]. Minor structural differences from coelenterazine may increase the specificity for human albumin. Attempts are underway to modify coelenterazine to develop a molecule that reacts more strongly with albumin [27]. However, there have been no attempts to apply it to medical measurement purposes, such as the measurement of albumin in urine.

As depicted in Figure 5, when the albumin was heat-denatured, the albumin–luciferin reaction significantly reduced the intensity of luminescence. By contrast, the immunoturbidimetric method did not reduce the signal, even with heat-denatured albumin. When the albumin was partially degraded by trypsin, the albumin–luciferin reaction was more susceptible, and the luminescence tended to decrease. These results indicate that *Watasenia* luciferin reacts with intact albumin but is less likely to react with partially degraded albumin. Specifically, the state of albumin recognized by *Watasenia* luciferin is slightly different from that recognized using the immunoturbidimetric and HPLC methods.

Among the protein precipitation methods evaluated in this study, the acetone method demonstrated the best correlation with the results of the immunoturbidimetric method (Figure 7D). This may be because most proteins were precipitated by the acetone method, and foreign substances were successfully removed. The ammonium sulfate and PEG methods may have incompletely removed the coexisting molecules, or the albumin may not have been precipitated completely. Acetone precipitation is often used for the pretreatment of proteomes [28] and is seldom used with the expectation of protein activity after precipitation because it is an organic solvent. A surprising finding was that the ability of the binding to luciferin was retained after acetone precipitation. Although acetone precipitation was successful in this study, centrifugation is a time-consuming process and must be improved if a simpler process is available.

When the results of the immunoturbidimetric method were compared with those of the *Watasenia* luciferin method after acetone precipitation, a correlation (R = 0.880) was obtained (Figure 7D); however, the *Watasenia* luciferin method tended to produce lower values. This is consistent with the fact that *Watasenia* luciferin is less likely to react with denatured or half-degraded albumin compared with the immunoturbidimetric method (Figure 5). We found that the luciferin method using acetone precipitation yields lower values compared to immunoturbidimetry, especially in the urine that contains high concentrations of albumin (Figure 8). Urine with high albumin concentrations may have a high percentage of half-degraded or modified albumin.

Currently, there is a lack of a perfect method for quantifying urinary albumin concentrations. Techniques such as the immunoturbidimetric assay, which is based on the antigen–antibody reaction, cannot detect peptide albumin and do not detect immuno–unreactive albumin [20]. However, the HPLC method, which is based on the principle of fractionation by molecular weight, can detect immuno–unreactive albumin, but it has the possibility of measuring proteins other than albumin that have similar molecular weights. The luciferin-based detection method we have developed in this study also tends to specifically detect intact albumin but not degenerated albumin. As these methods differ in terms of the state of albumin they detect, multiple methods of measurement can provide detailed information regarding the state of albumin in the urine. Urinary albumin has various states, such as intact, immuno–unreactive, peptide albumin, and dimers, and studies have reported that these states change according to the disease state [29,30,31,32]. A combination of multiple methods may help in clarifying the relationship between urinary albumin status and disease.

The luciferin-based albumin assay is a simple method based on the reaction with a single compound. This is an advantage, and there is no such thing as differences in antibody reactivity due to differences in immunized animals. If a method to purify urinary protein in a short time is developed, it is expected to become a simple and stable method for measuring urinary albumin.

## 4. Materials and Methods

### 4.1. Synthesis of Watasenia Luciferin

To obtain the desired *Watasenia* luciferin **1**, we synthesized pyrazin-2-amine **5** and acetal derivative **8**, according to a known method [33]. Commercially available pyrazine **2** was converted into dibromopyrazine **3** by the treatment of N-bromosuccinimide in dimethylsulfoxide and H_2_O. The Negishi cross-coupling reaction of **3** yielded the benzyl derivative **4**, which was further transformed into the desired pyrazin-2-amine **5** by the Suzuki–Miyaura coupling reaction in the presence of a catalytic amount of (PhCN)_2_PdCl_2_ and 1,4-bis(diphenylphosphino)butane (dppb) as a ligand, as shown in Scheme 1.

The phenolic hydroxyl group of p-hydroxybenzaldehyde **6** was protected with a tert-butyldimethylsilyl (TBS) group, followed by a reduction of the resulting TBS ether with NaBH_4_ to produce the benzyl alcohol derivative **7 [34]**. The benzyl chloride derivative derived from **7** was then converted into the corresponding Grignard reagent, which had to be further reacted with ethyl diethoxyacetate at −78 °C to obtain the desired acetal **8**. The Grignard reagent was prepared according to the method described by Shrestha and Bossmann et al. [33], but the desired acetal derivative **8** was seldom obtained (7% over two steps). However, when the Grignard reagent was prepared in the presence of 1,2-dibromoethane, 75% of the desired acetal derivative **8** was synthesized over two steps, as shown in Scheme 2.

After obtaining the desired pyrazin-2-amine **5** and acetal derivative **8**, we examined the synthesis of **1** according to the method described by S. Kojima et al., as shown in Scheme 3 [23]. The mixture of **5** and **8** in EtOH and 10% HCl solution were refluxed to yield 65% of the 3,7-dihydroimidazo[1,2a]pyrazine-3-one derivative **9**. Finally, the sulfonation of **9** was performed with sulfur trioxide pyridine complex (SO_3_-pyridine), followed by treatment with sodium methoxide to form the sodium salt of **1**. However, the desired sodium salt of **1** could not be obtained, and only a complex mixture was obtained (Scheme 3).

In 2003, M. Adamczyk et al. proposed a mechanism for luminescence via anion B when a series of derivatives A with a 3,7-dihydroimidazo[1,2a]pyrazine-3-one skeleton are deprotonated, as shown in Supplementary Figure S1 [35]. We believed that the failure to obtain the sodium salt of **1** was due to the decomposition of the corresponding bis-sulfonate anion of **9** by the addition of sodium methoxide, and we decided to attempt other sulfonation reaction conditions. We attempted the synthesis of **1** under acidic conditions, noting that the 3,7-dihydroimidazo[1,2a]pyrazine-3-one derivative **9** was obtained from **5** and **8** in a moderate yield after refluxing with 10% HCl solution. By treating **9** with an excess amount of chlorosulfonic acid, the desired **1** was successfully synthesized with a 65% yield (Scheme 4 and Supplementary Figure S2).

See the supplementary methods for more information.

### 4.2. Urine Samples and Other Materials

Urine samples were collected from 20 patients with diabetes from the University of Toyama Hospital and stored at −20 °C. Before usage, the urine samples were centrifuged at 17,800× *g* for 5 min to remove insoluble components.

The proteins (1 mg/mL) shown in Figure 1 consisted of papain (Wako), trypsin (Nacalai), RNaseA (Roche), transferrin (Nacalai), xanthine oxidase (Nacalai), collagenase (Worthington Biochemical), dispase (Thermo Fisher), proteinase K (Roche), human serum albumin (Nacalai), and bovine serum albumin (Sigma). IgG, shown in Figure 1, was raised in our laboratory [36].

### 4.3. Measurement of Luminescence

The intensity of luminescence was measured using a multiplate reader ARVO X2 (PerkinElmer). The solution used in the reaction consisted of 80 mM Tris (pH 10), 200 mM H_2_O_2_, 200 μM *Watasenia* luciferin, and 1 mg/mL protein sample (Figure 1, Figure 2 and Figure 5), which was performed in a 96-well plate (Nunclon Delta white microwell, Nunc). Spectrum measurements were performed using SpectraMax i3 (Molecular Device). The intensity of luminescence was measured at wavelengths at every 20 nm width. The solution consisted of 80 mM Tris (pH 10), 200 mM H_2_O_2_, 200 μM *Watasenia* luciferin, and 1 mg/mL human albumin.

### 4.4. Immunoturbidimetric Assay

N-assay TIA Micro Alb (Nittobo) was used to measure the albumin concentration in urine samples via immunoturbidimetry. According to the manufacturer’s instructions, 3.5 μL of urine samples and standards was mixed with 150 μL of buffer R1 in a 96-well plate (BM6000, BM Bio) and incubated at 37 °C for 5 min. Then, 50 μL of buffer R2 was added to the mixture and incubated at 37 °C for 5 min. Absorbance was measured at wavelengths of 700 and 340 nm using a SpectraMax i3 multiplate reader. The signal intensity of the sample was obtained by the subtraction of the OD value at 700 nm from that at 340 nm. Purified human albumin (Nacalai) was used as a standard.

### 4.5. Heat Inactivation and Trypsin Digestion of Human Albumin

Human albumin was heat-inactivated using a heat block (Zymoreactor II, ATTO) for 1, 2, 5, 10, and 30 min at 95 °C, and then it was immediately cooled on ice. For trypsin treatment, purified albumin was reacted with trypsin (Nacalai) at various concentrations (0%, 2.5 × 10^−2^%, 1.25 × 10^−3^%, 2.5 × 10^−3^%, and 2.5 × 10^−4^%) for 30 min at 37 °C. After the completion of the reaction, the albumin was immediately reacted with luciferin, and the luminescence was measured. The same samples were subjected to immunoturbidimetric assays to measure OD.

### 4.6. Acetone Precipitation

A mixture of 100 µL of the patient urine sample and 900 µL of the ice-cold acetone was cooled overnight at −20 °C. Next, the mixture was centrifuged at 17,800× *g* for 30 min, and the resulting precipitate was dissolved in 50 µL of PBS. Purified human albumin was treated in the same manner to be used as a standard.

### 4.7. Ammonium Sulfate Precipitation

Saturated ammonium sulfate solution (900 µL) was mixed with 100 µL of the urine sample and allowed to stand on ice for 1 h. It was then centrifuged at 17,800× *g* for 30 min, and the resulting precipitate was dissolved in 50 µL of PBS.

### 4.8. PEG Precipitation

PEG 8000 (MP Biomedicals) was dissolved in water to prepare a 50% (*w*/*w*) solution. Then, 100 μL of the PEG solution was mixed with 100 μL of the urine sample, placed on ice for 1 h, and centrifuged at 17,800× *g* for 30 min to precipitate the protein. The precipitate was dissolved in PBS and used for the reaction with luciferase.

### 4.9. Spike-Recovery Test

HSA (100 μg) was added to the patient urine (100 μL), acetone-precipitated, and reacted with luciferin to measure luminescence. The difference between the luminescence of urine with and without HSA was calculated. The same procedure was performed with PBS, and the recovery rate was calculated. This confirmed whether the components in the urine inhibited the reaction of HSA with luciferin.

### 4.10. Statistical Analysis

One-way ANOVA with Dunnett’s test or Tukey’s test was conducted to determine the statistical significance. The data were expressed as the mean ± SEM. *p* < 0.05 was considered to indicate statistical significance. In Figure 3 and Figure 7, the approximate straight line was computed by the least-squares method.

## 5. Conclusions

*Watasenia* luciferin specifically reacts with albumin and emits light in proportion to its concentration. We demonstrated that this luminescence can be used as an indicator to detect urinary albumin after acetone precipitation. This is a new principle for quantifying urinary albumin.

## Data Availability

The data presented in this study are available on request from the corresponding author.

## Supplementary Materials

The following supporting information can be downloaded at: https://www.mdpi.com/article/10.3390/ijms23158342/s1.
