# Supplementary material for: A New Method for Albuminuria Measurement Using a Specific Reaction between Albumin and the Luciferin of the Firefly Squid Watasenia scintillans
Source: Int J Mol Sci. 2022 Jul 28;23(15):8342. doi: 10.3390/ijms23158342 (PMC9368953; doi:10.3390/ijms23158342)
Supplement: Supplementary file 1 [file ijms-23-08342-s001.zip › ijms-1795377-SI.pdf]

Supplementary Materials

Supplementary Figures

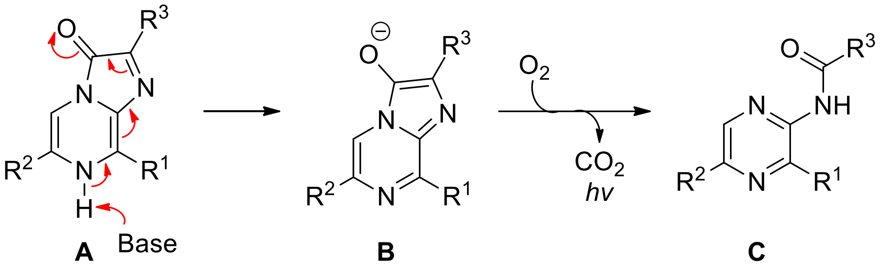

Supplementary Figure S1

Proposed mechanism for luminescence of 3,7-dihydroimidazo[1,2a]pyrazine-3-ones

^{1}H-NMR (400 MHz, CD_3OD)

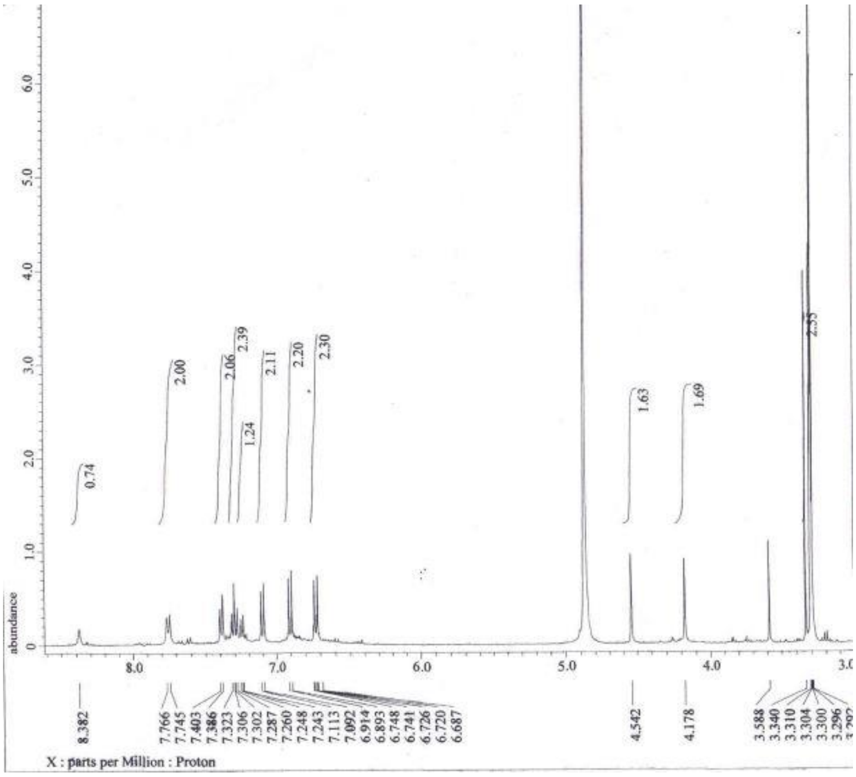

^{1}H-^{1}H COSY (400 MHz, CD_3OD)

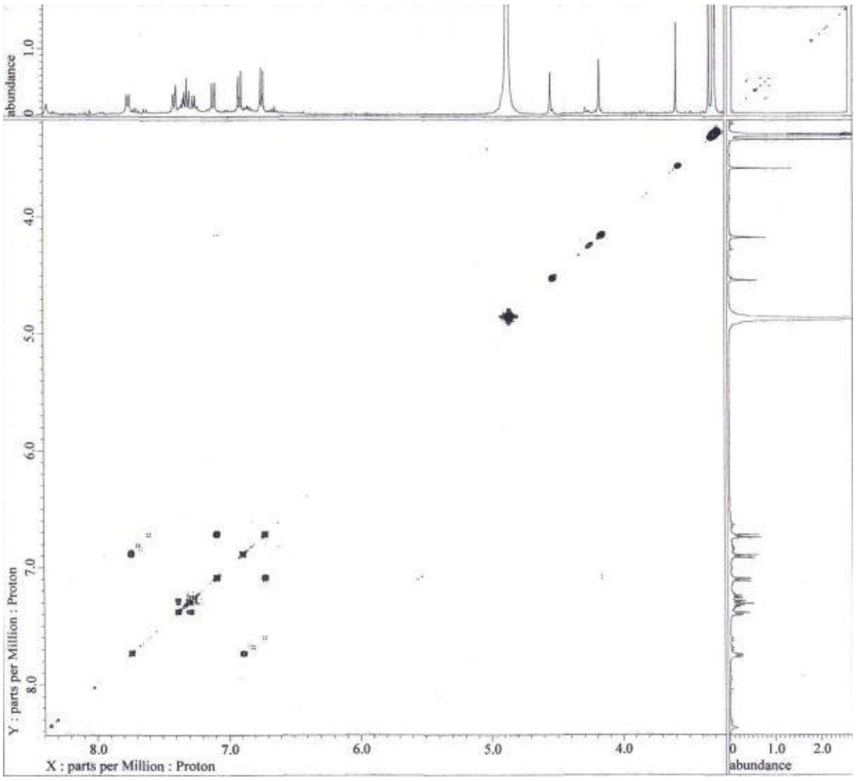

Supplementary Figure S2

NMR spectral data of *Watasenia* luciferin **1**

Supplementary methods

Chemistry

General information

Chemicals were purchased from Sigma-Aldrich, Merck (Darmstadt, Germany), FUJIFILM Wako Chemicals (Osaka, JAPAN), Nacalai Tesque, Tokyo Chemical Industry (Tokyo, Japan), and Kanto Chemical (Tokyo, Japan) and used without further purification. Column chromatography was done on Cica silica gel 60N (spherical,

neutral; particle size, 63–210 nm, Kanto Chemical) while thin-layer chromatography (TLC) was performed using Merck silica gel $60F_{254}$ plates. The nuclear magnetic resonance (NMR) spectra were acquired in the specified solvent in JEOL JNM-A400 (400 MHz for ^1H) or JEOL JNM-ECX500 (500 MHz for ^1H). The chemical shifts (δ) are reported in ppm downfield from TMS, and coupling constants (*J*) are expressed in Hertz.

*Synthesis of 3,5-dibromopyrazin-2-amine (**3**)*

To a stirred solution of pyrazin-2-amine **2** (500 mg, 5.26 mmol) in DMSO (15 mL) and H_2O (1 mL) was added NBS (1.96 g, 11.04 mmol) at 0 °C, and the resulting mixture was stirred at room temperature for 8 h. The reaction mixture was quenched with H_2O (5 mL). The aqueous mixture was extracted with EtOAc (5 mL x 3). The organic extracts were combined, dried, and evaporated to give a yellow oil, which was chromatographed on SiO_2 (20 g, *n*-hexane/acetone = 2/1) to give **3** (1.07 g, 4.26 mmol, 81%) as a white solid.

^1H-NMR (400 MHz, $CDCl_3$) δ: 8.02 (1H, s), 5.09 (2H, br); R_f = 0.60 (*n*-hexane/acetone = 2/1).

*Synthesis of 3-benzyl-5-bromopyrazin-2-amine (**4**)*

To a stirred solution of $ZnCl_2$ (389 mg, 2.86 mmol) in Et_2O (3 mL) were added BnMgCl, prepared from the activated magnesium (64 mg, 2.62 mmol) and BnCl (0.34 mL, 2.98 mmol) in THF (3 mL), $Pd(PPh_3)_2Cl_2$ (42 mg, 0.06 mmol), and **3** (150 mg, 1.19 mmol) at room temperature, and the resulting mixture was stirred at room temperature for 3 days. The reaction mixture was quenched with H_2O (3 mL). The layers were separated and the aqueous layer was extracted with EtOAc (2 mL x 3). The organic layer and extracts were combined, dried, and evaporated to give pale yellow oil, which was chromatographed on SiO_2 (20 g, *n*-hexane/acetone = 2/1) to give **4** (194 mg, 0.74 mmol, 62%) as pale yellow oil.

^{1}H-NMR (400 MHz, $CDCl_3$) δ: 8.00 (1H, s), 7.30-7.18 (5H, m), 4.43 (2H, br), 4.05 (2H, s); R_f = 0.55 (*n*-hexane/acetone = 2/1).

*Synthesis of 3-benzyl-5-(4-((tert-butyldimethylsilyl)oxy)phenyl)pyrazin-2-amine (**5**)*

To a stirred solution of **4** (41 mg, 0.16 mmol) in toluene (2 mL) and EtOH (0.5 mL) were added $(PhCN)_2PdCl_2$ (3 mg, 0.01 mmol), dppb (4 mg, 0.01 mmol), (*p*-TBSO)$C_6H_4B(OH)_2$ (50 mg, 0.20 mmol), and Na_2CO_3 (aq.) (0.5 mL, 1 M in H_2O) at room temperature, and the resulting mixture was refluxed for 15 h. The aqueous mixture

was extracted with EtOAc (1 mL x 3). The organic extracts were combined, dried, and evaporated to give a brown oil, which was chromatographed on SiO_2 (10 g, *n*-hexane/EtOAc = 2/1) to give **5** (59 mg, 0.15 mmol, 98%) as pale yellow solid.

^{1}H-NMR (400 MHz, $CDCl_3$) δ: 8.30 (1H, s), 7.79 (2H, d, J = 8.4 Hz), 7.30-7.23 (5H, m), 6.90 (2H, d, J = 8.4 Hz), 4.36 (2H, br), 4.15 (2H, s), 0.99 (9H, s), 0.20 (6H, s); R_f = 0.50 (*n*-hexane/EtOAc = 1/1).

Synthesis of (4-((tert-butyldimethylsilyl)oxy)phenyl)methanol (7)

To a stirred solution of *p*-hydroxybenzaldehyde **6** (1 g, 8.19 mmol) in CH_2Cl_2 (20 mL) were added imidazole (669 mg, 9.83 mmol), TBSCl (1.48 g, 9.83 mmol), and DMAP (50 mg, 0.41 mmol) at room temperature, and the resulting was stirred at room temperature for 20 h. The reaction mixture was quenched with sat. NH_4Cl (aq.) (15 mL). The layers were separated and the aqueous layer was extracted with CH_2Cl_2 (3 mL x 3). The organic layer and extracts were combined, dried, and evaporated to give a colorless oil, which was directly used in the next step. To a stirred solution of the above TBS ether in MeOH (20 mL) was added $NaBH_4$ (465 mg, 12.29 mmol) at 0 °C, and the resulting mixture was stirred at room temperature for 30 min. The reaction mixture was quenched with sat. NH_4Cl (aq.) (10 mL). The aqueous mixture was extracted with

EtOAc (5 mL x 5). The organic extracts were combined, dried, and evaporated to give a colorless oil, which was chromatographed on SiO_2 (10 g, *n*-hexane/EtOAc = 5/1) to give **7** (1.89 g, 7.94 mmol, 97% in 2 steps) as a colorless oil.

^{1}H-NMR (400 MHz, $CDCl_3$) δ: 7.23 (2H, d, J = 8.0 Hz), 6.82 (2H, d, J = 8.0 Hz), 4.59 (2H, s), 0.98 (9H, s), 0.20 (6H, s); R_f = 0.40 (*n*-hexane/EtOAc = 5/1).

*Synthesis of 3-(4-((tert-butyldimethylsilyl)oxy)phenyl)-1,1-diethoxypropan-2-one (**8**)*

To a stirred solution of **7** (434 mg, 1.82 mmol) in CH_2Cl_2 (7 mL) was added $SOCl_2$ (0.20 mL, 2.73 mmol) at 0 °C, and the resulting mixture was stirred at 0 °C for 2 h. The reaction mixture was quenched with sat. $NaHCO_3$ (15 mL). The layers were separated and the aqueous layer was extracted with CH_2Cl_2 (2 mL x 3). The organic layer and extracts were combined, dried, and evaporated to give a colorless oil, which was directly used in the next step. To a stirred solution of the above benzyl chloride derivative in THF (7 mL) was added the activated magnesium (234 mg, 9.65 mmol) and 1,2-dibromoethane (0.83 mL, 9.65 mmol) at room temperature, and the reaction mixture was heated at 50 °C for 1 h. A solution of ethyl diethoxyacetate (0.42 mL, 2.37 mmol) in THF (5 mL) was added to the reaction mixture at -78 °C, and the resulting mixture was stirred at -78 °C for 16 h. The reaction was quenched with sat. NH_4Cl (aq.) (5 mL).

The layers were separated and the aqueous layer was extracted with EtOAc (1 mL x 3). The organic layer and extracts were combined, dried, and evaporated to give a colorless oil, which was chromatographed on SiO_2 (20 g, *n*-hexane/EtOAc = 5/1) to give **8** (481 mg, 1.37 mmol, 75% in 2 steps) as a colorless oil.

^{1}H-NMR (400 MHz, $CDCl_3$) δ: 7.04 (2H, d, J = 8.8 Hz), 6.76 (2H, d, J = 8.8 Hz), 4.60 (1H, s), 3.78 (2H, s), 3.72-3.64 (2H, m), 3.55-3.47 (2H, m), 1.24-1.18 (6H, m), 0.95 (9H, s), 0.16 (6H, s); R_f = 0.65 (*n*-hexane/EtOAc = 5/1).

*Synthesis of 8-benzyl-2-(4-hydroxybenzyl)-6-(4-hydroxyphenyl)imidazo[1,2a]pyrazin-3(7H)-one (**9**)*

To a stirred solution of **5** (26 mg, 0.07 mmol) and **8** (47 mg, 0.13 mmol) in EtOH (3 mL) was added 10% HCl (aq.) (0.3 mL) at room temperature, and the resulting mixture was refluxed for 24 h. The solvent was evaporated to give a red oil, which was chromatographed on SiO_2 (8 g, *n*-hexane/EtOAc = 5/1) to give **9** (18 mg, 0.04 mmol, 65%) as a red solid.

^{1}H-NMR (400 MHz, CD_3OD) δ: 7.60 (1H, s), 7.46 (2H, d, J = 8.4 Hz), 7.37 (2H, d, J = 8.4 Hz), 7.30-7.17 (3H, m), 7.14 (2H, d, J = 8.4 Hz), 6.86 (2H, d, J = 8.4 Hz), 6.68 (2H, d, J = 8.4 Hz), 4.39 (2H, s), 4.06 (2H, s); R_f = 0.80 (CH_2Cl_2/MeOH = 5/1).

*Synthesis of Watasenia Luciferin (**1**)*

To a stirred solution of **9** (5 mg, 0.01 mmol) in MeCN (1.2 mL) and pyridine (0.3 mL) was added chlorosulfonic acid (8 μL, 0.12 mmol) at 0 °C, and the resulting mixture was stirred for 16 h. The solvent was evaporated to give a red solid, which was chromatographed on SiO_2 (3 g, *n*-hexane/EtOAc = 5/1) to give **1** (7 mg, 0.01 mmol, 65%) as a red solid.

^{1}H-NMR (400 MHz, CD_3OD) δ: 8.38 (1H, s), 7.76 (2H, d, *J* = 8.4 Hz), 7.39 (2H, d, *J* = 8.4 Hz), 7.30 (2H, t, *J* = 8.4 Hz), 7.25 (1H, t, *J* = 8.4 Hz), 7.10 (2H, d, *J* = 8.4 Hz), 6.90 (2H, d, *J* = 8.4 Hz), 6.73 (2H, d, *J* = 8.4 Hz), 4.54 (2H, s), 4.18 (2H, s); R_f = 0.55 (CH_2Cl_2/MeOH = 5/1).
